# Crushes of the Extremities

**Published:** 1908-05

**Authors:** Thos. H. Hancock

**Affiliations:** Atlanta, Ga.


### CRUSHES OF THE EXTREMITIES.*

BY THOS. H. HANCOCK, M. D., ATLANTA, GA.

These accidents may occur in the country or small towns, and the rule is to take them to one of the cities. This may be a good procedure in many cases where the loss of blood has not been great and the shock is not marked, but even the crushes of the arm, fore-arm or foot should not be neglected, as many of them have proved fatal.

Since we may have such an accident out in the country entirely away from a physician, the layman should know how to control hemorrhage. Constriction of the limb just above the injury is easily made by a piece of bell cord, a suspendor or a handkerchief. The mistake usually made is that they are not tight enough, and a moderate compression increases the venous flow. These improvised tourniquets become very painful, even when they are applied near the crushed area, *and may add to the shock, if left too long.* Whiskey *in moderation* will be a great help until

*Read before Medical Association of Georgia, Fitzgerald, April 15, 16, 17, '08

the physician arrives, when he can give a few whiffs of chloroform, if he knows how to give it; or ether, if he does not, as all doctors can give ether. Then he can easily cut away the mangled tissue and tie the most of the bleeding vessels. If he is embarrassed by any bleeding points which he cannot catch, he has simply to thread a curved needle with a piece of catgut and take a stitch through the tissues in such a manner as to include the vessel. (I have tied the superficial palmar arch in this way). The hemorrhage having been controlled, the wound should be irrigated with an antiseptic solution and dressed with gauze wet with a 1-3000 bichloride solution, and the ordinary dressing applied over it as a temporary dressing.

Now, after extensive crushes we have to fight against shock. The body must not only be treated but also the mind, and we can do much by reassuring them. The pain can be relieved by sulphate of morphia, which in small doses is a stimulant, but in large doses depresses. The dose should be from one-eighth to one-quarter of a grain hypodermically, and it may be repeated at intervals of an hour, if necessary. I have seen two cases that I am satisfied were killed by too large doses of morphine; and in one of the cases the physician acknowledged having given a grain hypodermically, as he said "the patient was restless and very hard to control." The restlessness never kills.

The head should be lowered and hot water bags or bottles (the latter can always be had) should be put around the body, which should be well covered. There is little use in putting medicine in the stomach, as it will probably be vomited. Warm salt solution thrown into the bowel does equally as well, if not better, than that given by infusion. An enema consisting of a pint of the normal salt solution to which has been added an ounce or two of whiskey and several ounces of black coffee will be found to be very effectual. Larger amounts of the solution are not so apt to be retained. The patient should be kept quiet, and should not be moved any considerable distance if shock is marked. The amputation may be postponed for several days if necessary.

This is one of the conditions; the other is that of the patient who has been injured close to a hospital where everything is in readiness for an immediate operation; both thighs possibly have been crushed, but in a short time he is on the operating table. The morphia (1-8 of a grain) and enema have been given. The anæsthetic has been commenced, and in less than an hour the patient is in a bed with the foot elevated, the hot water bags are about him and reaction soon begins to take place. The last two cases I have seen of double crushes of the thighs have recovered, and in both cases two of us operated at the same time. Drainage should always be used and an iodoform gauze dressing will prevent the early decomposition of the secretions. It should be changed, however, in twenty-four hours.

Three grains of the mild chloride of mercury should be given as soon as reaction has been established, and this should be followed in eight hours by half an ounce of Epsom salts, which should be repeated every four hours till the bowels have acted well. Should the third dose prove ineffectual, an enema consisting of half an ounce of fee bovis and half a pint of glycerine with a quart of warm water should be given. The diet is an important factor.

If he is in a hospital, he will be put on a liquid diet, but he does not always get it, as milk is on this list. Unmodified sweet milk drunk by the glass curds in the stomach in a large curd which is hard to digest and predisposes to flatulence. It is almost universally given and I mention it only to condemn it. If it is drunk through a nipple or a small pipette, very slowly, as suggested by Dr. Harris, it may be very good. Many of its modifications are very valuable, and especially is this so of malted milk and many of the creamed broths. Mixed vegetable soups containing meat fats are also bad. Various meat broths, albumens and other prepared foods may be had which will give variety and will answer every purpose.

His whiskey should be kept up indefinitely, if he has been a drinking man; and if he is a cigarette smoker, pity him and let him have cigarettes in moderation. Also, if he has any drug habit, reduce it gradually. The small things in our work often decide the result.

---

The thirteenth annual meeting of the Association of Surgeons of the Southern Railway Company was held at the Hillman Hotel, Birmingham, Ala., April 28, 29 and 30, 1908.

